# Characterization of pulmonary vascular remodeling and MicroRNA-126-targets in COPD-pulmonary hypertension

**DOI:** 10.1186/s12931-022-02267-4

**Published:** 2022-12-15

**Authors:** Khushboo Goel, Nicholas Egersdorf, Amar Gill, Danting Cao, Scott D. Collum, Soma S. Jyothula, Howard J. Huang, Maor Sauler, Patty J. Lee, Susan Majka, Harry Karmouty-Quintana, Irina Petrache

**Affiliations:** 1 Department of Medicine, Division of Pulmonary and Critical Care Medicine, National Jewish Health, Denver, USA; 2 Department of Medicine, Division of Pulmonary Sciences and Critical Care, University of Colorado, Aurora, USA; 3 Nova Southeastern University Dr. Kiran C. Patel College of Allopathic Medicine, Fort Lauderdale, USA; 4 Department of Biochemistry and Molecular Biology, University of Texas Health Science Center Houston, Houston, USA; 5 Department of Internal Medicine, Division of Pulmonary, Critical Care, and Sleep Medicine, University of Texas Health Science Center at Houston, McGovern Medical School, Houston, USA; 6 Division of Pulmonary Critical Care, Transplant Pulmonology, Houston Methodist Hospital, Houston, USA; 7 Department of Medicine, Division of Pulmonary, Allergy, and Critical Care Medicine, Yale School of Medicine , New Haven, USA; 8 Department of Medicine, Division of Pulmonary, Allergy, and Critical Care Medicine, Duke University School of Medicine, Durham, USA; 9 Divisions of Critical Care, Pulmonary and Sleep Medicine, Department of Internal Medicine, and Department of Biochemistry and Molecular Biology, McGovern Medical School, University of Texas Health Science Center at Houston, Houston, USA

**Keywords:** Chronic obstructive pulmonary disease, Emphysema, Endothelial cells, Smoking, Pulmonary vascular remodeling, Group 3 pulmonary hypertension, microRNA-126, ADAM9

## Abstract

**Background:**

Despite causing increased morbidity and mortality, pulmonary hypertension (PH) in chronic obstructive pulmonary disease (COPD) patients (COPD-PH) lacks treatment, due to incomplete understanding of its pathogenesis. Hypertrophy of pulmonary arterial walls and pruning of the microvasculature with loss of capillary beds are known features of pulmonary vascular remodeling in COPD. The remodeling features of pulmonary medium- and smaller vessels in COPD-PH lungs are less well described and may be linked to maladaptation of endothelial cells to chronic cigarette smoking (CS). MicroRNA-126 (miR126), a master regulator of endothelial cell fate, has divergent functions that are vessel-size specific, supporting the survival of large vessel endothelial cells and inhibiting the proliferation of microvascular endothelial cells. Since CS decreases miR126 in microvascular lung endothelial cells, we set out to characterize the remodeling by pulmonary vascular size in COPD-PH and its relationship with miR126 in COPD and COPD-PH lungs.

**Methods:**

Deidentified lung tissue was obtained from individuals with COPD with and without PH and from non-diseased non-smokers and smokers. Pulmonary artery remodeling was assessed by ⍺-smooth muscle actin (SMA) abundance via immunohistochemistry and analyzed by pulmonary artery size. miR126 and miR126-target abundance were quantified by qPCR. The expression levels of ceramide, ADAM9, and endothelial cell marker CD31 were assessed by immunofluorescence.

**Results:**

Pulmonary arteries from COPD and COPD-PH lungs had significantly increased SMA abundance compared to non-COPD lungs, especially in small pulmonary arteries and the lung microvasculature. This was accompanied by significantly fewer endothelial cell markers and increased pro-apoptotic ceramide abundance. miR126 expression was significantly decreased in lungs of COPD individuals. Of the targets tested (*SPRED1, VEGF, LAT1, ADAM9*), lung miR126 most significantly inversely correlated with *ADAM9* expression. Compared to controls, ADAM9 was significantly increased in COPD and COPD-PH lungs, predominantly in small pulmonary arteries and lung microvasculature.

**Conclusion:**

Both COPD and COPD-PH lungs exhibited significant remodeling of the pulmonary vascular bed of small and microvascular size, suggesting these changes may occur before or independent of the clinical development of PH. Decreased miR126 expression with reciprocal increase in ADAM9 may regulate endothelial cell survival and vascular remodeling in small pulmonary arteries and lung microvasculature in COPD and COPD-PH.

**Supplementary Information:**

The online version contains supplementary material available at 10.1186/s12931-022-02267-4.

## Introduction

Chronic obstructive pulmonary disease (COPD) is a top 4 leading cause of death [1–3]. Despite being characterized by only mild to moderate elevations in mean pulmonary artery pressure (mPAP), pulmonary hypertension (PH) is a major determinant of increased morbidity and mortality in COPD [4–7]. In fact, a 10 mmHg increase in mean pulmonary artery pressure at rest is associated with a fourfold increased mortality [8]. Unlike major advances in idiopathic pulmonary arterial hypertension (PAH), there is little understanding of the pathogenesis of PH in COPD (COPD-PH), explaining the paucity of therapeutic options for this condition.

Similar to PAH, pulmonary arteries in COPD-PH display hypertrophy of both intima and the vascular smooth muscle cells comprised tunica media [9], along with pruning of small pulmonary arteries and precapillary microvasculature [10]. However, unlike PAH, COPD-PH lungs do not exhibit hyperproliferative endothelial cells that form vascular occlusive plexiform lesions typical of PAH [11], suggesting that mechanisms of pulmonary vascular remodeling in COPD-PH may not fully recapitulate those of PAH.

Cigarette smoking (CS), the major etiological factor of COPD, causes endothelial cell injury and dysfunction, which are thought to be important processes in the pulmonary vascular remodeling in COPD-PH [7, 12], but a unifying mechanism that explains how pulmonary vascular beds of various size are differentially affected is currently lacking.

We recently identified that microRNA-126 (miR126), a well-established pro-angiogenic, pro-survival, and reparative master regulator of endothelial cells lining the large pulmonary and systemic vasculature [13], inhibits the migration, proliferation, and survival of human lung microvascular endothelial cells (HLMVEC) [14]. These differential effects on specific vascular beds are exerted by cell type-specific targets, whereby pro-angiogenic effects in large pulmonary artery endothelial cells are mediated by inhibition of SPRED1 (sprout related EVH1 domain-containing 1) or PIK3R2, both negative regulators of VEGF (vascular endothelial growth factor) signaling [15, 16]. In contrast, the miR126 anti-angiogenic effect in HLMVEC is mediated by inhibition of the amino acid transporter SLC7A5/LAT1 (solute carrier family 7 member 5), which is required for mTOR signaling [14]. The differential effect on endothelial cells from distinct pulmonary vessel sizes renders miR126 as a potential mediator in COPD-PH, but how miR126 and its targets are affected in COPD-PH has not been described.

Both CS exposure and COPD have a marked effect on miR126. CS exposure decreases the intracellular expression of miR126 in HLMVEC and increases the secretion of miR126 in endothelial exosomes [17]. Peripheral blood endothelial cells of smokers and COPD individuals, and lungs of mice exposed to CS [18] exhibit significantly decreased miR126 expression, as well. The functional effect of reduced miR126 in these conditions is not well elucidated. We found that the decrease in miR126 in CS-exposed HLMVEC may be adaptive, by protecting against apoptosis and by promoting tube formation, via increasing LAT1 [14]. In addition, we identified additional miR126 targets in HLMVEC including ADAM9. The metalloprotease ADAM9 has been recently recognized as an important mediator of not only acute lung injury, but also COPD, being implicated in lung inflammation, alveolar septal apoptosis, emphysema, small airway fibrosis, and mucous cell metaplasia in mice [19, 20]. In turn, in lung cancer, ADAM9 increases angiogenesis and enhances pulmonary vascular remodeling [19]. Together, these findings suggest that miR126 and its target ADAM9 may play an important role in the vascular remodeling in COPD, but their abundance in COPD-PH has not been reported yet.

Using human lung tissue from individuals with a history of smoking, COPD, or COPD-PH, we sought to characterize pulmonary arterial remodeling in these groups in relationship to miR126 and ADAM9 expression.

## Materials and methods

### Human lung tissue (Additional file 1: Table S1)

Deidentified lungs from non-diseased non-smokers and chronic smokers were obtained from National Jewish Health Human Lung Consortium; lungs from Global Initiative for Chronic Obstructive Lung Disease (GOLD) stage 4 COPD were obtained from Lung Tissue Research Consortium (LTRC); and lungs from COPD and COPD-PH were obtained from University of Texas Houston Lung Biobank.

### Immunohistochemistry

Sections of 3 µm thickness from paraffin-embedded lungs were heated to 60 °C, followed by de-paraffinization in CitriSolv Hybrid Solvent (Thermo Fisher; 04-355-121; Waltham, MA, USA) and serial washes of 100%, 95%, and 70% ethanol solutions. Antigen retrieval was performed in a pressure cooker with citric acid-based Antigen Unmasking Solution (Vector; H-3300; Burlingame, CA, USA; 1:100). The tissue sections were then incubated in 0.3% hydrogen peroxide (Sigma-Aldrich; St. Louis, MO, USA; H325-100) for 1 h, blocked in horse serum (Vector; PK-6200; 1:100), incubated with smooth muscle actin (SMA) primary antibody (Dako; M0851; Glostrup, Denmark; 1:500) overnight at 4 °C, followed by biotinylated anti-mouse secondary antibody (Vector; BA-2000; 1:250) for 1 h at room temperature. The technical negative control was lung tissue stained exclusively with secondary antibodies. The sections were then incubated with avidin–biotin complex (Vector; PK-4000) followed by DAB substrate (Vector; SK-4100). The sections were mounted with Aqua Poly mounting media (Pleasances; 18606-100; Warrington, PA, USA). Blinded to the identity of the slides, images were captured at 10 × using a Nikon 80i microscope and 6 random quadrants per slide were analyzed using ImageJ software (NIH).

### Quantification of pulmonary vascular remodeling and pulmonary artery classification

Using ImageJ software, the outer border of the tunica media and border of the artery lumen was manually outlined and the respective cross-sectional areas within these borders were measured. The proportion of the pulmonary artery staining positive for SMA was quantified as: SMA area/(SMA area + arterial lumen area). Because these lungs were not processed in a manner to allow for stereologic measurements, pulmonary arteries were classified by size based on their cross-sectional areas (Table 1): medium (> 31,415.9 µm^2^), small (3848.45–31,415.9 µm^2^), and microvascular (< 3848.5 µm^2^). The ranges of cross-sectional areas were extrapolated using the equation $\pi {r}^{2}$, where r = diameter/2, based on published external diameter ranges [21, 22]: medium (200–500 µm), small (70–200 µm), or microvascular (< 70 µm).

**Table 1:** Pulmonary artery classification

Pulmonary artery classification	Diameter	Cross sectional area
Medium	200–500 µm	> 31,415.9 µm^2^
Small	70–200 µm	3848.5–31,415.9 µm^2^
Microvascular	< 70 µm	< 3848.5 µm^2^

The cross-sectional area of each pulmonary artery was measured using ImageJ, using the external border of the tunica media as the outer edge. Three size classifications based on the cross-sectional area were created: medium (> 31,415.9 µm^2^), small (3848.5–31,415.9 µm^2^), and microvascular (< 3848.5 µm^2^). The ranges of cross-sectional areas were extrapolated using the equation $\pi {r}^{2}$, where r = diameter/2, based on published external diameter ranges [21, 22]: medium (200–500 µm), small (70–200 µm), or microvascular (< 70 µm)

### Immunofluorescence

De-paraffinization and antigen retrieval were performed as described for immunohistochemistry. The sections were blocked with 10% normal goat serum (Vector; S-1000-20) and 0.1% Triton-X solution (Sigma-Aldrich; × 100–100 mL) in PBS and incubated with primary antibodies overnight at 4 °C: mouse anti-ceramide (Enzo Life Sciences; ALX-804-196-T050; Farmingdale, NY, USA; 1:50), rabbit anti-CD31 (Novus Biologicals; NB 100-2284; Littleton, CO, USA; 1:200), rabbit anti-ADAM9 (Abcam; Ab186833; Cambridge, UK; 1:70), and/or mouse anti-CD31 (Cell Signaling Technology; CS3528S; Danvers, MA, USA; 1:250). Sections were then incubated with their corresponding secondary antibodies: goat anti-rabbit conjugated to Alexa Fluor 568 (Invitrogen; A11036; Waltham, MA, USA; 1:250) and goat anti-mouse conjugated to Alexa Fluor 488 (Invitrogen; A21121; 1:250). The technical negative control was lung tissue stained exclusively with secondary antibodies. TrueView Autofluorescence Quenching Kit was applied per manufacturer’s instructions (Vector; SP-8500-15). Sections were mounted with Prolong Gold with DAPI mounting media (Thermo Fisher; P36931). Images were captured at 20X using a Nikon 80i microscope (5 random quadrants per slide) and analyzed blinded to the identity of the slides using ImageJ2 software (Fiji).

### Lung genomics research consortium (LGRC) cohort

We re-analyzed the microRNA microarray profiles of flash frozen parenchymal lung tissues performed by the LGRC, focusing on miR126. Lung tissue samples were obtained from the NHLBI-sponsored LTRC (GSE72967, GSE47460) representing  explanted, resected, or biopsied lung tissue of individuals with and without COPD. Methods of tissue procurement, cohort characteristics, and gene expression profiling have been previously described [23].

### RNA isolation, reverse transcription, and RTqPCR

RNA isolation was performed on frozen lung tissue using the RNEasy Plus Micro kit (Qiagen; 74034; Germantown, MD, USA) or miRNeasy Tissue/Cells Advanced Mini Kit (Qiagen; 217604) following the manufacturer’s instructions. Reverse transcription followed by RTqPCR was performed on the StepOnePlus System (software version 2.3) using Taqman gene expression assays. The following primers were used: miR126-3p (Thermo Fisher; 4427975; assay ID 002228; target sequence UCGUACCGUGAGUAAUAAUGCG), miR126-5p (Thermo Fisher; 4427975; assay ID 000451; target sequence CAUUAUUACUUUUGGUACGCG), SPRED1 (Thermo Fisher; Hs01084559_m1), VEGFA (Thermo Fisher; Hs00900055_m1), LAT1 (Thermo Fisher; Hs01001189_m1), and ADAM9 (Thermo Fisher; Hs00177638_m1). Human 18S (H18S) (Thermo Fisher; 4333760F) was used as the housekeeping gene for mRNA quantification and RNU48 (Thermo Fisher; 4427975; assay ID 001006; target sequence GAUGACCCCAGGUAACUCUGAGUGUGUCGCUGAUGCCAUCACCGCAGCGCUCUGACC) was used for miRNA quantification. Relative RNA expression was quantified as 2^−∆∆CT^ as previously described [24].

### Cell culture

Primary HLMVEC were obtained from a commercial source (Lonza; CC2527; Basel, Switzerland) and maintained in complete culture medium EGM-2 (Lonza; CC3156) supplemented with its specific Bullet-Kit (Lonza; CC4147). All experiments were performed in HLMVEC between passages 3 to 7. The smoking status, age, and gender of the donor were provided by the supplier.

### miRNA transfection

 We used lysates from experiments previously performed and described by Cao et al. [14]. Briefly, HLMVEC from non-smoker donors were transfected at 80% to 90% confluency using lipofectamine RNAiMAX (Invitrogen; 13778150) with either miR126-3p-mimic (Dharmacon; C-300626-07-0002; Lafayette, CO, USA; 5 nM; 16 h), to achieve miR126 overexpression (OE); or with antisense miR126-3p (Dharmacon; IH-300626-08-0002; 5 nM; 16 h), to achieve miR126 knockdown (KD). Control cells were transfected with non-targeting (NT) mimics (Dharmacon; D-001810-10-05; 5 nM; 16 h). Transfection efficiencies were verified with real-time quantitative polymerase chain reaction (RTqPCR) and previously published by Cao et al. [14].

### Protein isolation and western blotting

To extract proteins, cells were incubated with lysis buffer comprised of RIPA buffer (Sigma-Aldrich; R0278), PhosStop (Sigma-Aldrich; 4906837001), and Complete (Sigma-Aldrich; 4693116001) tablets, which contain protease inhibitor cocktails, followed by sonication and centrifugation for 10 min at 4 °C to collect the supernatant. Equal protein amounts, as determined by the Pierce bicinchoninic acid assay protein analysis (Thermo Fisher; 23225; Waltham, MA, USA), were separated by SDS-PAGE and transferred onto a polyvinylidene difluoride membrane (EMD Millipore; IPLF10100; Burlington, MA, USA), followed by routine western immunoblotting. Blots were washed with TBS + 0.1% Tween-20 (TBST), blocked in TBST with 5% BSA solution, and incubated overnight with the primary antibody ADAM9 (Cell Signaling Technology; 2099S; 1:500–1:1000). Blots were then washed and incubated with rabbit HRP-conjugated secondary antibody (Sigma-Aldrich; NA9340V; 1:10,000). Immune complexes were detected using enhanced chemiluminescence, quantified by densitometry (ImageJ software) and normalized to vinculin (Abcam; Ab18058; 1:5000) as the loading control.

### Statistical analysis

Statistical analysis was performed using Prism (GraphPad; version 9.1.1; La Jolla, CA, USA). Comparisons among groups were made using 1-way ANOVA with Dunnett’s multiple comparisons tests, unpaired t-test, and Pearson’s correlation analysis, as appropriate. All data are expressed as mean ± SD or SEM, as appropriate. Statistical difference was accepted at a *P* value < 0.05.

## Results

### Pulmonary arterial remodeling in chronic smokers, COPD, and COPD-PH

**Fig. 1:**
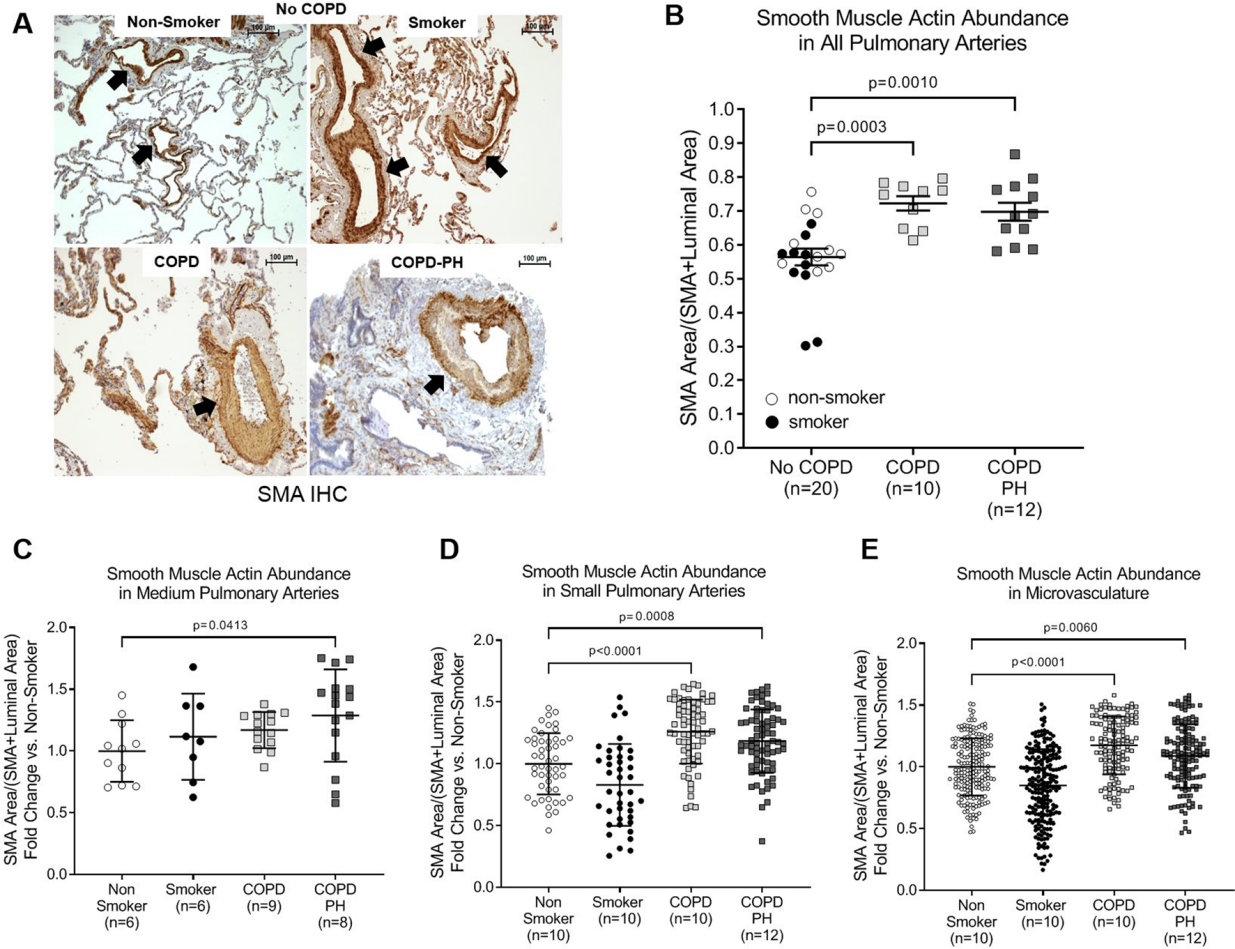
Pulmonary Arterial Remodeling in COPD and COPD-PH. **A** Representative images of smooth muscle actin (SMA) (brown) in human lung tissue from non-smokers and smokers without COPD as well as COPD and COPD-PH individuals, determined by immunohistochemistry (IHC). The slides were counterstained with hematoxylin (blue). Black arrows point to pulmonary arteries. Images were captured at 10X; scale bar is 100 µm. **B–E** Quantification of pulmonary arterial remodeling was performed by calculating the proportion of the artery which stained positive for SMA. **B** Quantification of SMA abundance in pulmonary arteries, regardless of size. Each data point represents an individual; horizontal lines are mean ± SEM. SMA abundance analyzed by size of: (**C**) medium pulmonary arteries, (**D**) small pulmonary arteries, and (**E**) microvasculature, with each data point representing a single pulmonary vessel and horizontal lines representing mean ± SD. For all graphs, n = number of individuals in each group; 1-way ANOVA with Dunnett’s multiple comparisons test was used for statistical analysis

Immunohistochemistry staining for SMA was performed on human lung tissue sections from non-smokers, chronic smokers without lung disease, COPD, and COPD-PH individuals to quantify the degree of pulmonary arterial remodeling in the tunica media and intima (Fig. 1A). Compared to non-smokers and smokers without COPD, lungs of COPD and COPD-PH had significantly increased SMA abundance in pulmonary arteries of all sizes (p = 0.0003 and 0.001, respectively) (Fig. 1B). We then assessed pulmonary arterial remodeling based on the pulmonary artery size classification. Interestingly, chronic smokers without COPD did not have significantly increased SMA abundance compared to non-smokers, apart from a slight relative increase in the medium sized pulmonary arteries (Fig. 1C). Consistent with the data for all pulmonary arteries, both COPD and COPD-PH individuals had significantly increased SMA abundance, particularly in the small pulmonary arteries (p < 0.0001 and 0.0008, respectively) and the microvasculature (p < 0.0001 and 0.006, respectively) (Fig. 1D, E). There was no significant correlation between the degree of pulmonary vascular remodeling and mean pulmonary artery pressure (mPAP) (Additional file 1: Fig. S1).

### Endothelial cell and pro-apoptotic ceramide abundance in pulmonary arteries

**Fig. 2:**
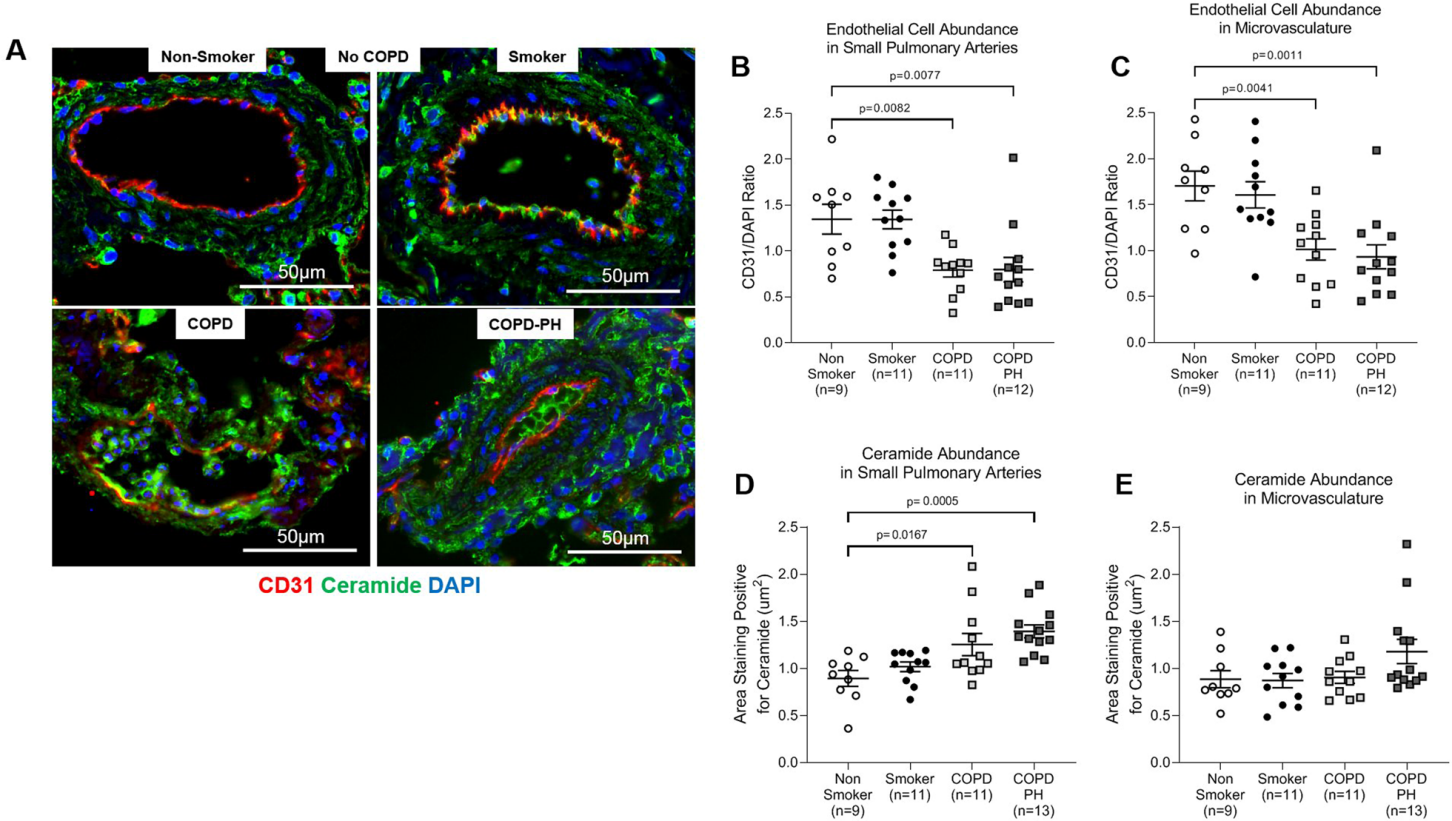
Endothelial cell and ceramide abundance in pulmonary arteries. **A** Representative images of immunofluorescence performed on human lung tissue from non-smokers and smokers without COPD as well as COPD and COPD-PH individuals, staining for the endothelial cell marker CD31 (red), ceramide (green), and DAPI (blue). Each image shows a pulmonary artery. Images were captured at 20×; scale bar is 50 µm. **B**, **C** Quantification of endothelial cell abundance in (**B**) small pulmonary arteries and (**C**) the microvasculature. **D**, **E** Quantification of ceramide abundance in (**D**) small pulmonary arteries and (**E**) the microvasculature. For all graphs, each data point represents an individual; n = number of individuals in each group; horizontal lines are mean ± SEM; 1-way ANOVA with Dunnett’s multiple comparisons test was used for statistical analysis

Since endothelial cell dysfunction and destruction of the vascular bed are two key mechanisms of pulmonary vascular remodeling in COPD and COPD-PH [7], we assessed the presence of endothelial cells in small pulmonary arteries and the microvasculature, which is where we identified the highest levels of remodeling. Using immunofluorescence to stain for the endothelial cell marker CD31 (Fig. 2A), we found that COPD and COPD-PH individuals had significantly decreased endothelial cell abundance in small pulmonary arteries (p = 0.008 each) and the microvasculature (p = 0.004 and 0.001, respectively) compared to non-smokers and chronic smokers without COPD (Fig. 2B, C). We then stained for ceramide, a bioactive sphingolipid causally implicated in lung endothelial cell apoptosis and emphysema [3], and found increased ceramide abundance in the COPD and COPD-PH groups, which reached statistical significance in small pulmonary arteries (p = 0.017 and 0.0005, respectively) (Fig. 2D, E). These findings suggest endothelial cell and pulmonary arterial injury in COPD and COPD-PH.

### Expression of miR126 and its targets in lung tissue

**Fig. 3:**
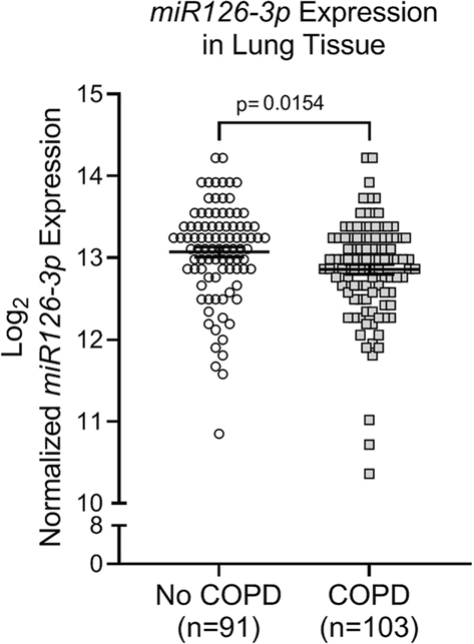
miR126 Expression in COPD. *MiR126-3p* expression from reanalysis of microarray profiles of human lung tissue from individuals with or without COPD performed by the Lung Genomics Research Consortium (LGRC). The samples were obtained from the NHLBI-sponsored Lung Tissue Research Consortium (LTRC) (GSE72967, GSE47460). Each data point represents an individual; horizontal lines are mean ± SEM; unpaired t-test was used for statistical analysis

Given the prominent role of miR126 in regulating pulmonary vascular endothelial cell survival and repair [13], we next quantified miR126 expression in human lung tissue. We analyzed pre-existing miRNA microarray profiles obtained by the LGRC and found that lung tissue from COPD individuals had significantly decreased miR126 expression compared to control lung tissue (p = 0.015) (Fig. 3).

**Fig. 4:**
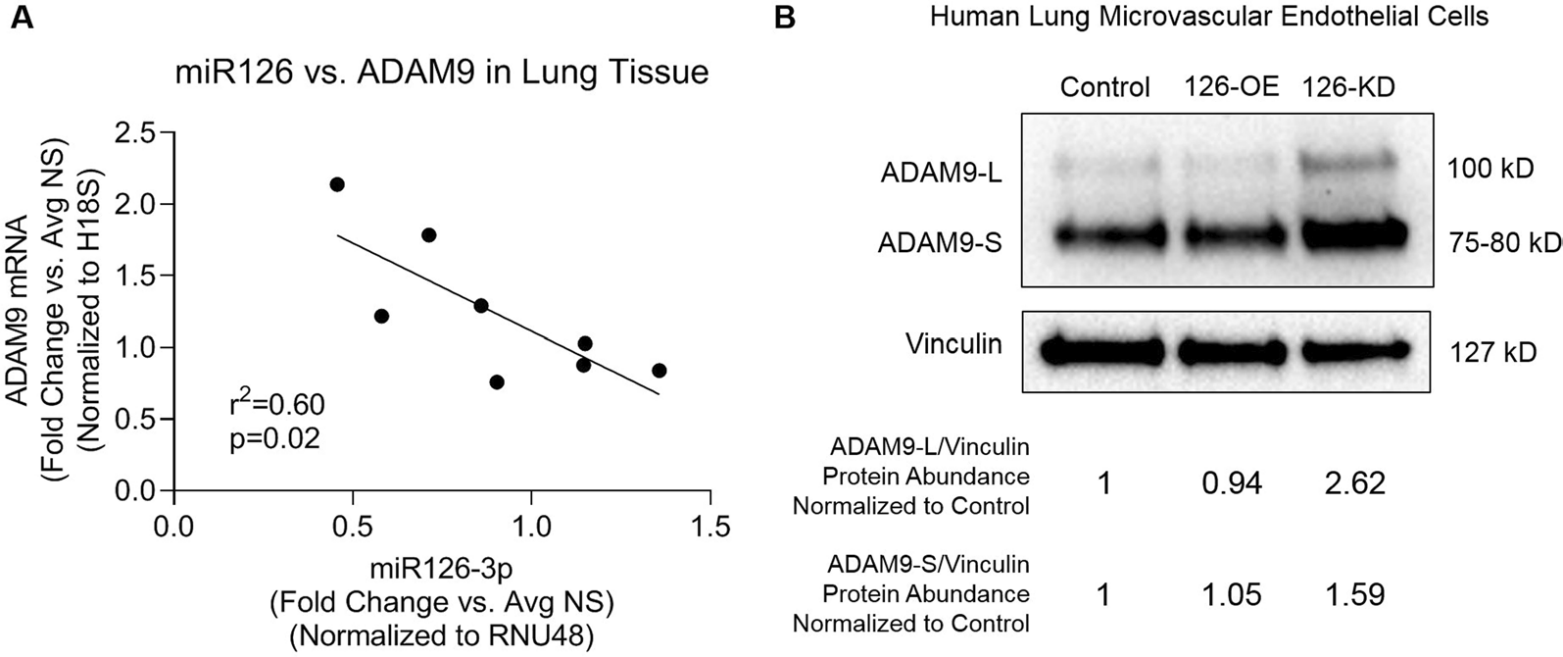
Correlation between miR126 and ADAM9. **A** Linear regression of association of lung *miR126* and *ADAM9* mRNA expression levels measured by RTqPCR in non-smokers (n = 4), smokers without COPD (n = 3), and COPD-PH (n = 1) individuals. Each data point represents an individual; Pearson’s correlation analysis was used for statistical analysis. **B** ADAM9 long (L) and short (S) isoforms abundance in western blots of human lung microvascular endothelial cells from non-smokers transfected with miR126-3p-mimic (126-OE) or antisense (126-KD), using vinculin as loading control. Below, normalized levels are quantified by densitometry

Building on our previously published data about miR126-targets in HLMVEC [14], we sought to identify which mRNA targets had a significant inverse correlation with miR126 in the lung tissue. We did not find any significant correlation between miR126 expression and *LAT1*, *SPRED1*, or *VEGFA* expression in human lung tissue from non-smokers, smokers, and COPD-PH individuals (Additional file 1: Fig. S2). In turn, *ADAM9* expression was significantly inversely correlated with miR126 expression in human lung tissue (r^2^ = 0.06, p = 0.02) from non-smokers, smokers, and COPD-PH individuals (Fig. 4A). We further validated this reciprocal relationship between miR126 and ADAM9 in HLMVEC, finding that miR126-KD resulted in increased ADAM9 protein abundance (Fig. 4B).

### ADAM9 abundance in pulmonary arteries

**Fig. 5:**
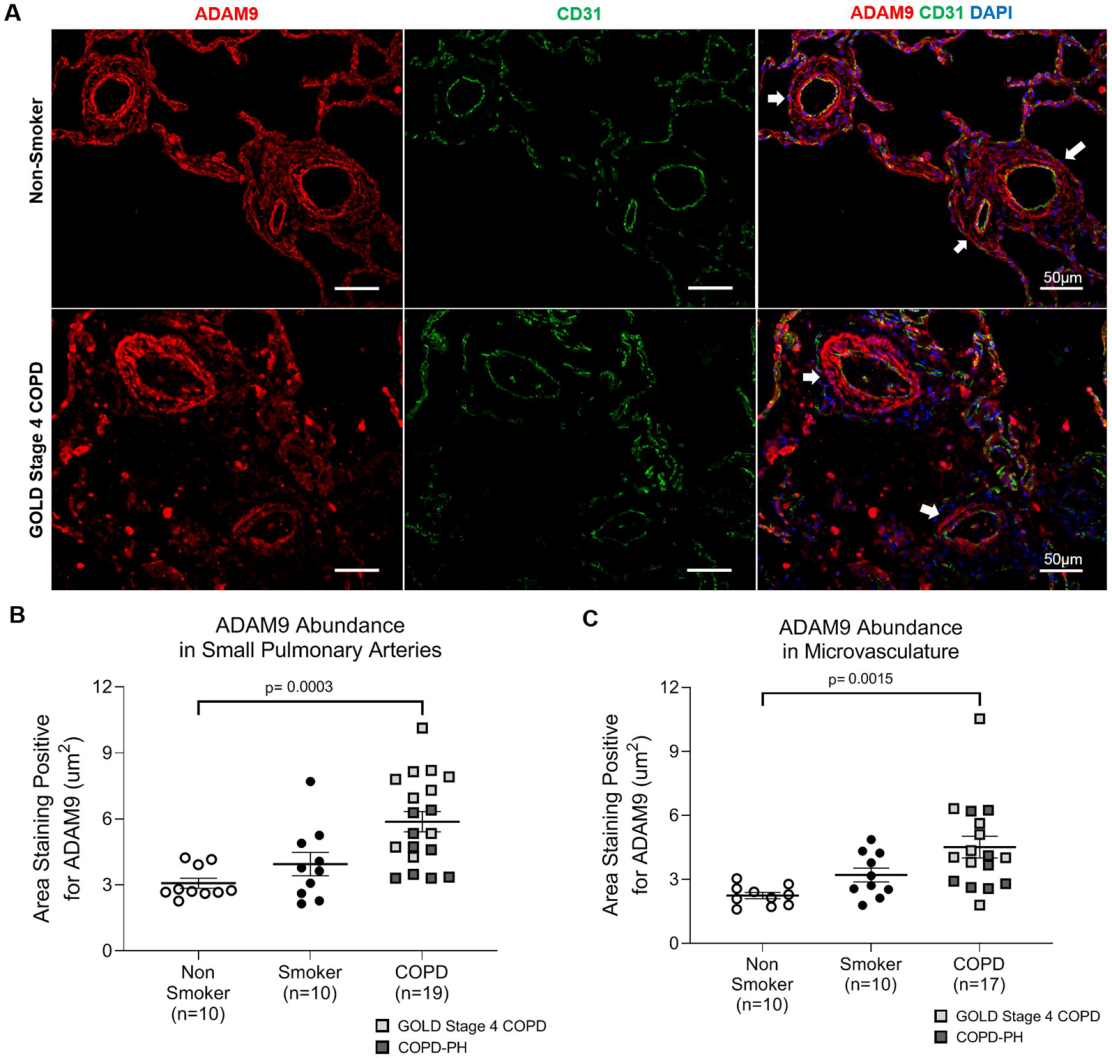
ADAM9 abundance in pulmonary arteries. **A** Representative images of ADAM9 (Red) endothelial cell marker CD31 (green), and DAPI (blue) abundance detected by immunofluorescence performed on human lung tissue from non-smokers and smokers without COPD, GOLD stage 4 COPD, and COPD-PH individuals. White arrows point to pulmonary arteries. Images were captured at 20X; scale bar is 50 µm. **B**, **C** Quantification of ADAM9 abundance in (**B)** small pulmonary arteries and (**C**) the microvasculature. For all graphs, each data point represents an individual; n = number of individuals in each group; horizontal lines are mean ± SEM; 1-way ANOVA with Dunnett’s multiple comparisons test was used for statistical analysis

Given our findings of decreased miR126 in COPD and an inverse correlation between miR126 and ADAM9, we hypothesized that the remodeled pulmonary vasculature in COPD will be characterized by increased ADAM9 abundance. We performed immunofluorescence staining for ADAM9 on a cohort of non-smokers, chronic smokers, GOLD stage 4 COPD (with unknown or undefined PH status), and COPD-PH individuals (Fig. 5A). We selected lungs from individuals with GOLD stage 4 COPD (FEV1 < 30%) because the prevalence of PH is as high as 90% in individuals with severe emphysema (90%) [4–7]. We found that individuals with severe COPD that are likely to have PH  as well as individuals with confirmed COPD-PH had significantly increased ADAM9 abundance in small pulmonary arteries (p = 0.003; Fig. 5B) and the microvasculature (p = 0.002; Fig. 5C).

## Discussion

We identified significantly increased remodeling of the small pulmonary arteries and microvasculature in all COPD lungs, regardless of mPAP. The endothelial cell-enriched miR126 is decreased in COPD lungs and is inversely correlated with *ADAM9* expression. Finally, we found that ADAM9 abundance is significantly increased in the small pulmonary arteries and microvasculature of COPD lungs, coupled with decreased endothelial cell- and increased pro-apoptotic ceramide abundance.

Our finding of significant morphological and endothelial cellular changes  in small pulmonary arteries and the microvasculature in COPD lungs without hemodynamic evidence of PH furthers our understanding of the characteristics of pulmonary vascular remodeling in COPD. Our data are consistent with prior work by Magee et al. who showed that patients with mild to moderate COPD without pulmonary hypertension develop intimal thickening [25]. Our finding is also compatible with the work of Bunel et al. who found that COPD patients with severe PH undergoing lung transplantation had an increased remodeling score of the microvessels compared to COPD patients with moderate PH or those without PH [26]. Interestingly, Santos et al. showed that even smokers without COPD develop intimal thickening and pulmonary arterial wall thickness [27], and Seimetz et al. showed that pulmonary vascular remodeling and pulmonary hypertension preceded the development of emphysema in mice exposed to cigarette smoke [28]. We did not find that smokers without COPD had significantly increased pulmonary arterial remodeling apart from a slight trend in medium sized pulmonary arteries. However, this may be due to differences in our donor patient population: the cohort of smokers without COPD had an average age of 47.1 years, which was at least 10 years younger than the COPD and COPD-PH cohorts and even younger than the non-smokers without COPD cohort (average age 50.4 years). One limitation of our methodology was that due to a limited number  lung tissue samples available, we performed IHC only for SMA, without cell type-specific co-staining, and therefore we were unable to identify whether the remodeling was due to intimal proliferation or medial hypertrophy.

The significant remodeling noted in the COPD-only cohort is consistent with the lack of a correlation between SMA abundance and mPAP. In fact, pulmonary arterial wall thickness, quantified with SMA abundance, has not been consistently correlated with mPAP, particularly in the absence of vaso-occlusive plexiform lesions [29–31]. Taken together and in context of published literature, our data indicate that significant remodeling of the pulmonary vascular bed of small and microvascular size occurs before or independent of the clinical development of PH. These findings highlight the importance of defining the mechanisms of pulmonary vascular injury and dysfunction which are associated with chronic smoking and COPD, rather than focusing solely on established COPD-PH.

Building on the work of Paschalaki et al. who found decreased miR126 in mouse lung tissue after 28 days of CS exposure [18], we found significantly decreased miR126 in human lung tissue from individuals with COPD compared to individuals without COPD. Although this finding does not capture the cell-specific quantification of miR126, it highlights a strong signal for overall decreased miR126 levels, which may have different downstream consequences in different endothelial cell-types. Although miR126, by playing a key role in pulmonary vascular endothelial cell survival and repair [13] is an attractive target for COPD-PH, other miRNAs may also be involved in the development of COPD and COPD-PH. For example, Hertig et al. found that miR125a, 130a, and 424-5p were significantly decreased in explanted lung tissue from COPD-PH patients and were inversely correlated with mPAP [32].

We focused on ADAM9, which we found was significantly inversely correlated with miR126 expression in human lung tissue, suggesting it is a direct target, which is consistent with other published work and publicly available datasets which predict biological targets of miRNA [14, 33–35]. Moreover, ADAM9 has emerged as a key player in the pathogenesis of COPD/emphysema [19, 20]. Similarly, Wang et al. showed that there was increased ADAM9 expression in mouse lungs exposed to CS and in the alveolar epithelium in individuals with COPD [20]. Although ADAM9 has been shown to enhance pulmonary vascular remodeling in lung cancer via VEGFA, angiopoietin-2, and tissue plasminogen activator [19, 36], there is a paucity of literature linking ADAM9 to PH. White et al. performed microarray analysis in pulmonary arteries and found a 1.39 fold change increase in ADAM9 expression in chronically hypoxic mice overexpressing serotonin transporter as a model of PAH, compared to control mice [37]. We found that ADAM9 abundance was significantly increased in the same pulmonary vascular beds which exhibited the most amount of remodeling of COPD and COPD-PH individuals (small pulmonary arteries and lung microvasculature). ADAM9 is ubiquitously expressed and secreted by multiple cell types [19]. For example, we noted abundant ADAM9 staining in the tunica media of the pulmonary arteries (comprised primarily of smooth muscle cells). While it is possible that ADAM9 is regulated by other miRNAs in smooth muscle cells, Shen et al. showed that miR126 negatively regulates ADAM9 in both human aortic endothelial and smooth muscle cells [34]. In addition, miR126 may be secreted by endothelial cells within exosomal cargo [17], or as shown by Zhou et al., within protein complexes that can be taken up by smooth muscle cells to alter gene expression [38, 39]. Future studies will have to elucidate the role of cell-specific and intercellular communication between miR126 and ADAM9 in pulmonary vascular remodeling.

Although our data await mechanistic validation, given the well phenotyped human samples studied and the corroborative biological plausible link with histological and biochemical markers of pulmonary vascular remodeling, our findings support the importance of further investigations into the role miR126 and ADAM9 in COPD-PH pathogenesis.

## Conclusion

Decreased miR126 expression in COPD may impact endothelial cell survival and subsequent vascular remodeling in small pulmonary arteries and lung microvasculature via ADAM9.

## Supplementary Information

**Additional file 1**: **Table S1**. Characteristics of human lung tissue donors. **Fig. S1**. Correlation between mPAP and pulmonary arterial remodeling. **Fig. S2**. Correlation between miR126 and mRNA Targets.

## Data Availability

The datasets used and/or analyzed during the current study are available from the corresponding author on reasonable request.

## Abbreviations

COPD: Chronic obstructive pulmonary disease
CS: Cigarette smoking
DLCO: Diffusion capacity for carbon monoxide
FEV1: Forced expiratory volume in 1 s
FVC: Forced vital capacity
GOLD: Global initiative for chronic obstructive lung disease
hAoEC: Human aortic endothelial cells
HASMC: Human aortic smooth muscle cells
HLMVEC: Human lung microvascular endothelial cells
IF: Immunofluorescence
IHC: Immunohistochemistry
LGRC: Lung Genomics Research Consortium
LTRC: Lung Tissue Research Consortium
miR126: MicroRNA-126
mPAP: Mean pulmonary artery pressure
PAH: Pulmonary arterial hypertension
PH: Pulmonary hypertension
SLC7A5/LAT1: Solute carrier family 7 member 5
SMA: Smooth muscle actin
SPRED1: Sprout related EVH1 domain-containing 1
VEGF: Vascular endothelial growth factor
