# Supplementary material for: Characterization of pulmonary vascular remodeling and MicroRNA-126-targets in COPD-pulmonary hypertension
Source: Respir Res. 2022 Dec 15;23:349. doi: 10.1186/s12931-022-02267-4 (PMC9756782; doi:10.1186/s12931-022-02267-4)
Supplement: Supplementary file 1 — Additional file 1: Table S1. Characteristics of human lung tissue donors. Fig. S1. Correlation between mPAP and pulmonary arterial remodeling. Fig. S2. Correlation between miR126 and mRNA Targets. [file 12931_2022_2267_MOESM1_ESM.docx]

**Additional file 1**

**Table S1**

**
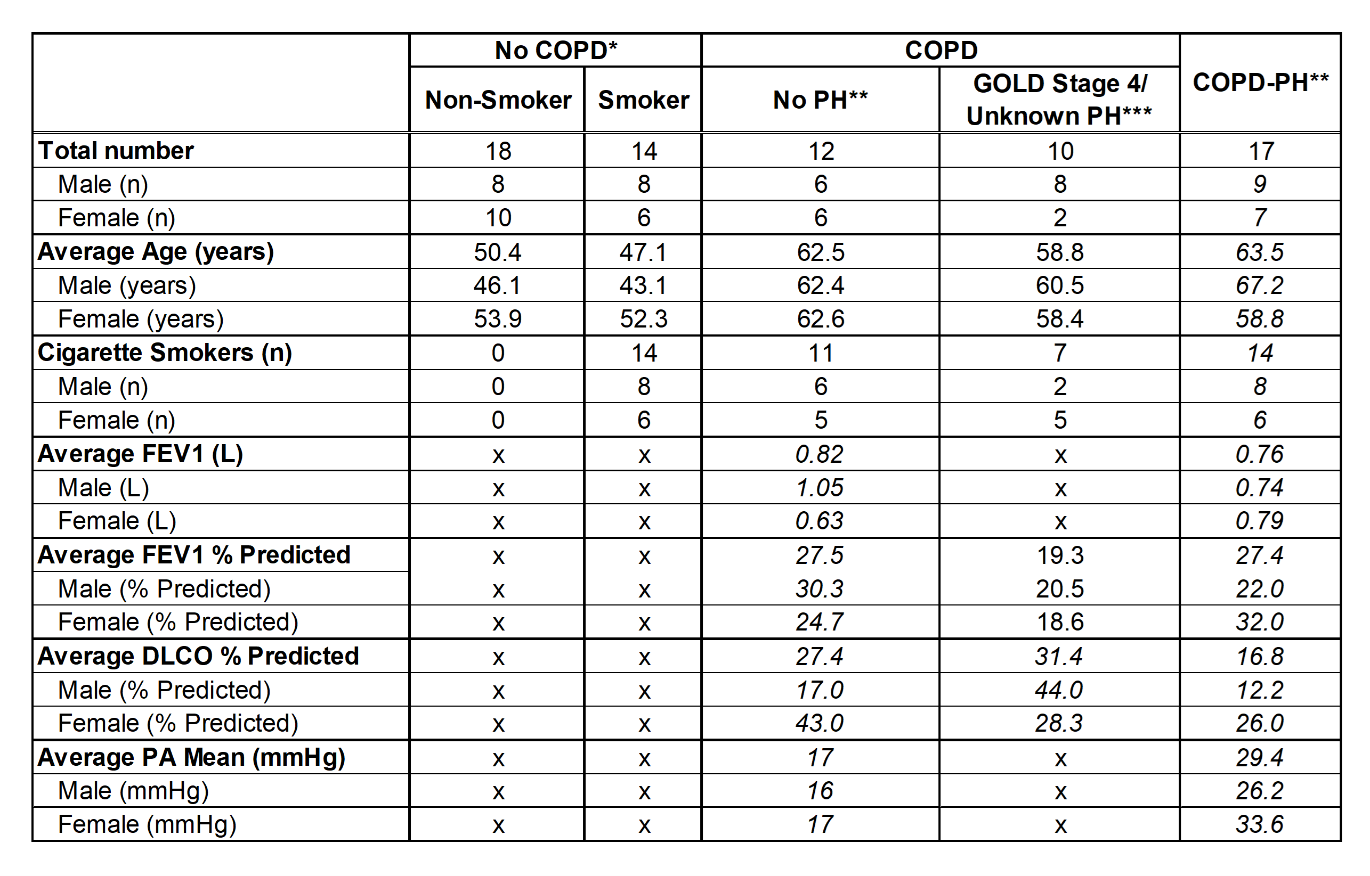
**

**Characteristics of Human Lung Tissue Donors.** Demographic and clinical information for the human lung tissue samples are listed: age, smoking history, forced expiratory volume in 1 second (FEV1), diffusion capacity for carbon monoxide (DLCO), and mean pulmonary artery pressure (mPAP). Non-smokers were defined as individuals without any smoking history or a very remote and minimal smoking history. Smokers were defined as individuals with active smoking history until their death. Individuals with COPD were defined as having an FEV1/FVC (forced vital capacity) ratio <0.70 and an FEV1 <80% predicted. The COPD-PH individuals met the definition for COPD and had a mPAP >20 mmHg, measured via right heart catheterization. *The non-smoker and smoker lung tissue was obtained from the National Jewish Health Human Lung Tissue Consortium; donors did not have underlying lung disease and died of a non-pulmonary cause. **Lung tissue from donors with COPD (of any GOLD staging) without PH and COPD-PH was obtained from the University of Texas Houston Lung Biobank. ***GOLD stage 4 lung tissue, defined as the donors having an FEV1 <30% predicted, was obtained from the Lung Tissue Research Consortium. X: Information is not available or not applicable. The italicized numbers represent values with missing data from their respective cohort.

**Figure S1**

**
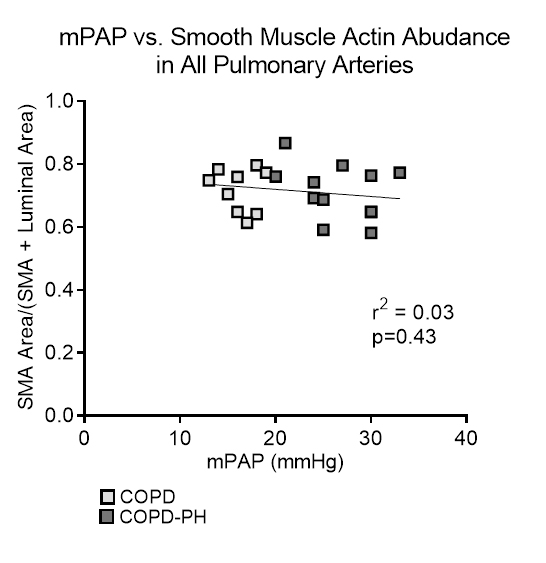
**

**Correlation between mPAP and Pulmonary Arterial Remodeling.**

Pulmonary arterial remodeling was quantified by measuring smooth muscle actin (SMA) abundance, as described in Figure 1. Mean pulmonary artery pressure (mPAP) was measured by right heart catheterization. Each data point represents an individual (COPD n=9, COPD-PH n=11); Pearson’s correlation analysis was used for statistical analysis.

**Figure S2**

**
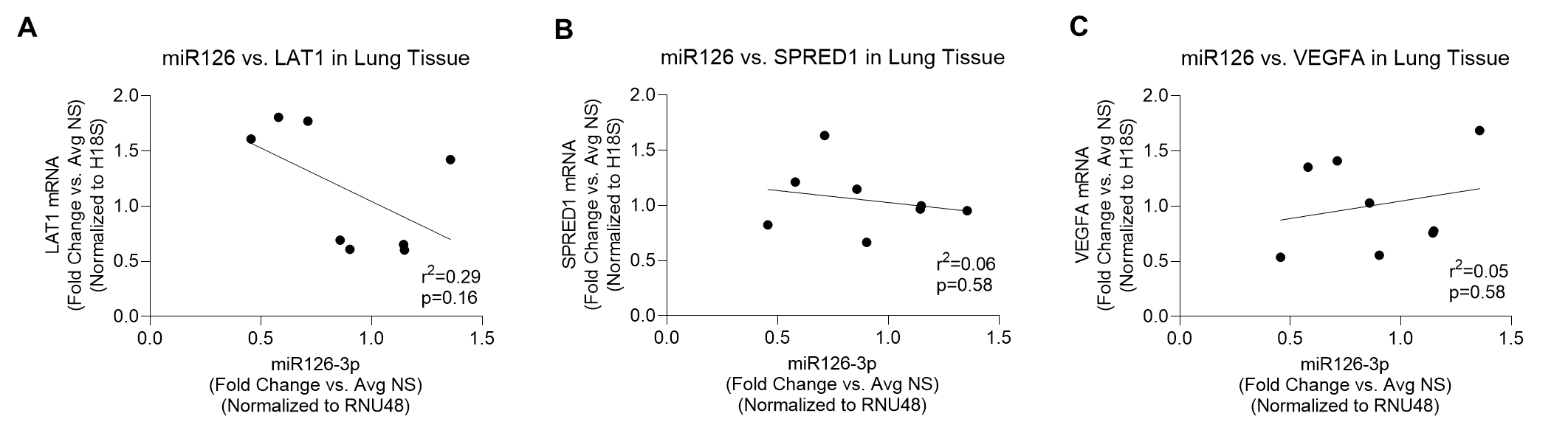
**

**Correlation between miR126 and mRNA Targets.** RNA was isolated from flash frozen human lung tissue from non-smokers and smokers without COPD and COPD-PH individuals, followed by RTqPCR to quantify *miR126* and *LAT1*, *SPRED1*, and *VEGFA* expression. Correlations are shown between miR126 and **(A)** LAT1, **(B)** SPRED1, and **(C)** VEGFA expression. For all graphs, each data point represents an individual; Pearson’s correlation analysis was used for statistical analysis.
